# Prevalence and impact of frailty on unplanned hospitalizations among adult patients with cancer in Taiwan

**DOI:** 10.1093/oncolo/oyaf234

**Published:** 2025-07-26

**Authors:** Chang-Hsien Lu, Kun-Yun Yeh, Yu-Shin Hung, Wen-Chi Chou

**Affiliations:** Department of Hematology and Oncology, Chang Gung Memorial Hospital at Chiayi, Chiayi 600, Taiwan; Department of Hematology and Oncology, Chang Gung Memorial Hospital at Keelung, Keelung 204, Taiwan; Department of Hematology and Oncology, Chang Gung University, Taoyuan 333, Taiwan; Department of Hematology and Oncology, Chang Gung University, Taoyuan 333, Taiwan; Geriatric Medical Center, Chang Gung Memorial Hospital at Linkou and College of Medicine, Chang Gung University, Taoyuan 333, Taiwan

**Keywords:** frailty, geriatric assessment, hospitalization, vulnerability

## Abstract

**Introduction:**

Frailty is common among older patients with cancer and may influence treatment outcomes. This study evaluated frailty prevalence and its association with unplanned hospitalizations in adult cancer patients.

**Materials and Methods:**

We prospectively included 2081 patients with newly diagnosed cancer from 3 medical centers in Taiwan (2018-2022) who were scheduled to undergo cancer treatment. Frailty was assessed using a comprehensive geriatric assessment within 7 days before treatment initiation. Patients were categorized as fit, pre-frail, or frail to assess the impact on 90-day unplanned hospitalization.

**Results:**

The prevalence of fit, pre-frail, and frail patients was 16.7%, 32.2%, and 51.0%, respectively. Frailty was associated with older age, unmarried status, lower education, unemployment, poor ECOG performance status, cancer site, and advanced stage (all *P* < .001). Core contributors to frailty across cancer sites included malnutrition, comorbidity, and polypharmacy. Functional impairment was prominent in patients with gastrointestinal, hematologic, and genitourinary cancers, while cognitive impairment was notable in those with genitourinary cancers. Unplanned hospitalization rates were highest in the frail group (41.5%), followed by pre-frail (34.4%) and fit (25.9%) patients. Frailty remained independently associated with unplanned hospitalizations after adjustment: pre-frail (adjusted odds ratio [OR] 1.44; 95% CI 1.06-1.94; *P* = .018) and frail (OR 2.07; 95% CI 1.55-2.76; *P* < .001).

**Conclusion:**

Frailty was prevalent and significantly associated with increased risk of unplanned hospitalization. These findings support the integration of frailty screening in routine oncology care to guide clinical decisions for vulnerable cancer patients.

Implications for Practice Frailty was identified in more than half of the adult cancer patients in this large, prospective cohort and was significantly associated with unplanned hospitalizations within 90 days of treatment initiation. These findings highlight the need to integrate routine frailty assessment into standard oncology workflows, even among non-elderly patients. Early identification of pre-frailty and frailty allows for risk stratification and timely interventions, potentially improving care coordination and reducing avoidable hospitalizations. Tailoring treatment decisions based on frailty status may also support better clinical outcomes and resource utilization across diverse cancer populations.

## Introduction

Frailty, a geriatric syndrome characterized by decreased physiological reserves and vulnerability to stressors, is common among older adults with cancer.^1^ Due to an aging population and rising prevalence of cancer, frailty has emerged as a critical determinant of health outcomes in patients with cancer.^2^^,^^3^ A large-scale systematic review of 2916 participants from 20 studies determined a median frailty prevalence of 42% (range 6%-86%) among older patients with cancer.^4^ Frailty has been associated with reduced tolerance, higher risk of treatment-related complications, and poorer overall survival outcomes in patients with cancer.^5^ Given the high prevalence of frailty and its connection to adverse survival outcomes, a routine frailty assessment is recommended for older patients with cancer to guide decisions about antitumor treatments.^6–8^

Taiwan’s aging population and the high cancer incidence underscore the urgent need for a comprehensive understanding of frailty.^9^ This includes determining frailty prevalence, identifying key domain deficits, and understanding the impact of frailty on treatment outcomes in older patients with cancer.^10^ Previous studies conducted in Taiwan have reported a frailty prevalence ranging from approximately 8.7% to 11.2% among community-dwelling individuals aged 65 years and older,^11–14^ which increases to 29.3%-44.7% in older adults with chronic illnesses.^15–17^ Despite extensive research on frailty in the general older population, its prevalence and impact in older Taiwanese patients with cancer remain poorly understood. Small studies have found this group’s frailty rates range from 29.5% to 55.6%.^18–21^ Frailty is also a significant and often overlooked issue for patients with cancer who are not older adults, with rates from 20% to 30%.^22–25^ The prevalence of frailty in patients with cancer varies widely and depends on factors, such as age, cancer site, treatment, and overall health.^26^ Although the link between frailty and cancer is well-established,^27^ gaps remain regarding its prevalence and impact across age strata and tumor types in Asian populations.

A consistent tool to assess frailty would enable meaningful comparisons of frailty prevalence and its impact across different cancer sites in the Taiwanese population. Accurately estimating the prevalence of frailty and identifying specific domain deficits in patients with cancer are critical steps toward designing targeted interventions to improve patient outcomes. This study addressed this gap by investigating the prevalence of frailty and identifying the key physiological factors that contributed to frailty among adult patients with various types of cancer in Taiwan. Although frailty is most commonly associated with biological aging, accumulating evidence indicates that it can manifest in younger adults with cancer through mechanisms, such as treatment‐related toxicity, chronic comorbid conditions, cancer cachexia, and psychosocial vulnerability.^22–25^ This study aimed to (1) determine the prevalence of frailty and pre-frailty in adult patients with cancer across different age groups and cancer sites; (2) identify the most frequently impaired frailty domains by cancer site; and (3) evaluate the association between frailty status and 90-day unplanned hospitalizations.

## Materials and methods

### Study design and data collection

This prospective observational cohort study enrolled consecutive patients newly diagnosed with cancer between 2018 and 2022 at 3 medical centers across Taiwan. Eligible participants were aged 20 years or older, had pathologically or radiologically confirmed cancer, and were planning to receive antitumor treatment. Patients were excluded if they were unable to complete the frailty assessment questionnaire or declined to provide written informed consent. The study protocol was approved by the Institutional Review Boards of the Chang Gung Memorial Hospitals at all participating sites (PMRPG3M0031) and has been conducted in compliance with the Helsinki Declaration (1996).

Sociodemographic variables included age, sex, marital status, education level, and employment status. Clinical variables included primary tumor site, cancer stage, Eastern Cooperative Oncology Group (ECOG) performance status, smoking history, alcohol consumption, and hospital site. Smoking and alcohol status were based on self-reported use within the past 12 months. ECOG PS was assessed by the treating oncologist at baseline.

### Frailty assessment

Frailty was assessed using a comprehensive geriatric assessment (CGA) that evaluated 8 domains: (1) functional status (Barthel Index and Lawton IADL), (2) comorbidity (Charlson Comorbidity Index), (3) cognition (Mini-Mental State Examination), (4) mood (Geriatric Depression Scale-15), (5) nutritional status (Mini Nutritional Assessment-short form, MNA-SF), (6) polypharmacy (≥5 medications daily), (7) social support (living status), and (8) falls history (≥2 falls in the past year).^18^ An abnormality in each domain was defined using validated cutoff criteria as detailed in Table S1. Frailty classification was based on the number of abnormal CGA domains, consistent with prior studies and population-specific validation in Taiwanese cancer patients.^25^^,^^28^ Fit was defined as 0 abnormal domains, pre-frail as 1, and frail as ≥2. This categorization reflects cumulative deficits and has been shown to predict adverse outcomes in oncology populations. Within 7 days prior to initiating anticancer therapy, a research assistant invited every eligible patient to complete an in-person CGA, which trained research nurses administered via structured, validated interviews, and corroborated through medical-record review.

### Primary outcome

The primary outcome was unplanned hospitalizations within 90 days after the CGA. To evaluate the primary outcome, the study team maintained close contact with the participants throughout the 90-day follow-up period and regularly reviewed their medical records to document any unplanned hospitalizations that occurred during that time.^21^ Hospitalizations were identified via the shared electronic medical record (EMR) network linking the 3 participating centers and confirmed during routine follow-up visits; however, because this platform does not extend beyond these institutions, admissions to outside hospitals were not captured and were therefore excluded from the analysis. Those with at least one unplanned hospitalization before death were counted as having the outcome. Patients who died without a hospitalization were classified as non-­hospitalized for this analysis.

### Subgroup analysis by age

To examine whether age modifies the predictive value of frailty, we conducted age-stratified multivariable logistic regression analyses (<65 vs ≥65 years), adjusting for sex, marital status, and tumor stage. We also visualized CGA dimension impairments by age group to explore age-specific patterns of frailty components.

### Statistical analyses

The characteristics of the study participants were summarized as numbers and percentages for the categorical variables. Differences in the categorical variables between frailty groups were compared using the Kruskal–Wallis test for continuous and ordinal variables and the chi-square or Fisher’s exact test for categorical variables. The odds ratio (OR) of the 90-day unplanned hospitalization rate was calculated using a logistic regression model. We used multivariable logistic regression to evaluate the relationship between frailty status and 90-day unplanned hospitalization. Age, sex, marital status, primary tumor site, and tumor stage were entered as covariates, chosen a priori for their clinical relevance and because previous evidence links these factors to hospitalization risk in oncology populations.^18–25^ To avoid over-adjustment, we excluded variables that were core components of the frailty definition (eg, ECOG performance status, nutritional status) from the primary multivariable model. However, we conducted a sensitivity analysis including ECOG performance and MNA-SF scores to assess the robustness of the association.

### Multivariable model and interaction testing

To evaluate potential effect modification, we tested key 2-way interaction terms among covariates in the final multivariable model, including age × sex, age × marital status, sex × marital status, and age × cancer site. None of these interactions reached statistical significance (all *P *>* *.1), and their inclusion did not materially affect the association between frailty and unplanned hospitalization. Covariate selection was guided by a priori knowledge, clinical relevance, and evidence from prior literature.^18–20^ To further clarify the hypothesized relationships among variables and inform our adjustment strategy, we constructed a directed acyclic graph, presented in Figure S1 (see online supplementary material for a color version of this figure).

### Sensitivity analysis for competing risk

To account for potential competing risk from early mortality, we conducted a sensitivity analysis excluding patients who died within 90 days of treatment initiation (*n* = 108, 5.2%). Although cause-specific competing risk models could not be applied due to data limitations, this exclusion-based approach provides a conservative estimate of frailty’s effect on unplanned hospitalization while minimizing survivor bias.

As the primary outcome was binary (presence or absence of unplanned hospitalization), we used standard logistic regression rather than ordinal logistic models. Therefore, testing of the proportional odds assumption was not applicable. Basic diagnostic checks confirmed model fit and absence of multicollinearity among covariates. SPSS software (version 22.0; IBM Corp., Armonk, NY, USA) was used for the statistical analyses. All statistical assessments were 2-sided, and a *P*-value <.05 was considered significant.

## Results

### Patients characteristics

**Figure 1.**
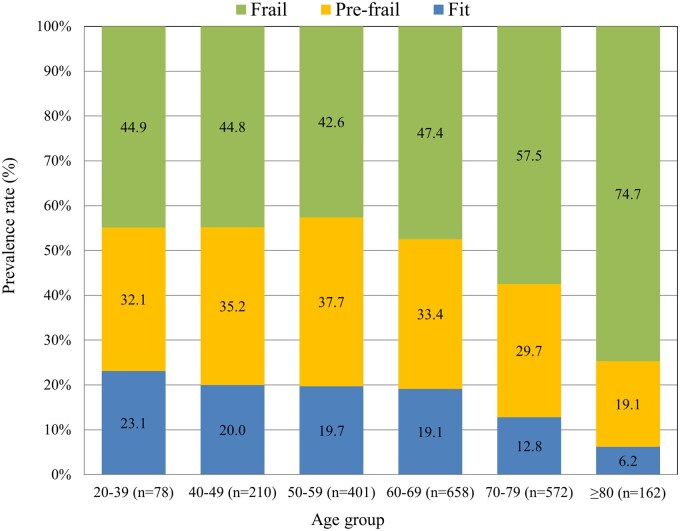
Frailty prevalence by age groups.

**Figure 2.**
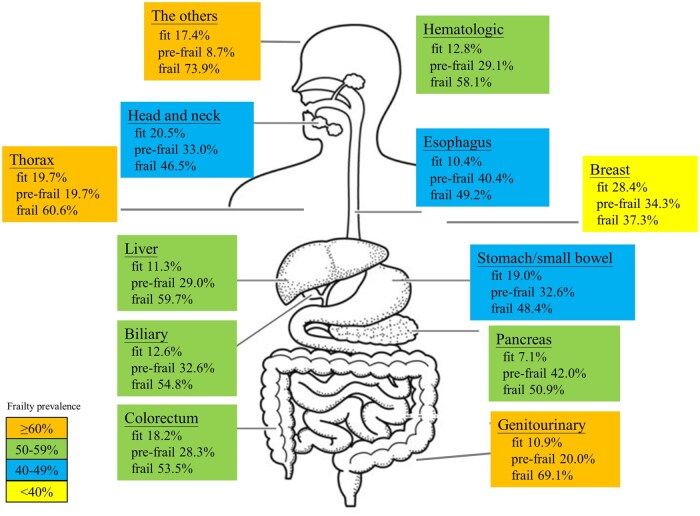
Frailty prevalence by cancer sites.

Of the 2081 participants, the median age was 65 years (IQR 55-75), and 2-thirds were men (67%). Most were married (79%), not currently employed or retired (56%), and had completed only primary or secondary education (81%). The distribution of cancer site, stage, and ECOG performance status is detailed in Table 1. Frailty categorization showed 348 patients (16.7%) were fit, 671 (32.2%) were pre-frail, and 1062 (51.0%) were frail. Frailty prevalence increased with age; the youngest group (20-39 years) had a 44.9% frailty rate, which was comparable to the next age group (40-49 years) rate of 44.8% (Table 1). The frailty prevalence increased more steeply in the older age groups, reaching 47.4% for those aged 60-69 years, 57.5% for those aged 70-79 years, and peaked at 74.7% for patients aged 80 years and older (Figure 1). The fit rate decreased with age; the highest rate was 23.1% in the 20-39 years group, and the lowest rate was 6.2% for patients aged 80 years and older. Sex differences were relatively minor; frailty was slightly more common among women than among men. Frailty was more common in unmarried patients (62%) than in those who were married (48.1%). Prevalence also varied by education, being highest among individuals with primary or secondary schooling and lowest among those with education beyond college. A similar pattern was seen with employment status: patients who were not working showed a higher proportion of frailty (56.2%) than those who were employed (43.8%). Frailty prevalence likewise raised with cancer stage, from 42% in stage I to 55.1% in stage IV. Frailty prevalence varied across cancer sites and is illustrated in Figure 2. The highest frailty rates were observed for patients with genitourinary cancer (69.1%) and “others” (73.9%), followed by those with thoracic cancer (60.6%). Moderate frailty prevalence (50%-59%) was observed in patients with liver (59.7%), hematologic (58.1%), biliary (54.8%), colorectal (53.5%), and pancreatic (50.9%) cancers. A lower frailty prevalence (40%-49%) was noted for those with esophageal (49.2%) and stomach/small bowel (48.4%) cancers, whereas patients with breast cancer had the lowest frailty prevalence (37.3%). Detailed patient characteristics stratified by cancer site are presented in Table S2.

**Table 1.** Frailty prevalence by patient demographics (*n* = 2081).

			Frailty status (%)			
Variable	Category	Overall, *n* (%)	Fit	Pre-frail	Frail	*P*-value
Age in years	20-39	78 (3.7)	23.1	32.1	44.9	<.001
	40-49	210 (10.1)	20.0	35.2	44.8	
	50-59	401 (19.3)	19.7	37.7	42.6	
	60-69	658 (31.6)	19.1	33.4	47.4	
	70-79	572 (27.5)	12.8	29.7	57.5	
	≥80	162 (7.8)	6.2	19.1	74.7	
Sex	Men	1397 (67.1)	17.2	33.5	49.3	.08
	Women	684 (32.9)	15.8	29.7	54.5	
Married status	Married	1641 (78.9)	18.0	33.9	48.1	<.001
	Unmarried	440 (21.1)	12.0	25.9	62.0	
Educational level	Nil or elementary school	707 (34.0)	15.6	26.6	57.9	<.001
	High school	986 (47.4)	15.7	35.3	49.0	
	College	355 (17.1)	20.6	35.2	44.2	
	Higher than college	33 (1.6)	30.3	30.3	39.4	
Current working status	Not working	1169 (56.2)	13.3	30.0	56.7	<.001
	Working	912 (43.8)	21.2	35.1	43.8	
Main care-giver	Partner/spouse	992 (47.7)	17.7	34.7	47.6	.011
	Other	1089 (52.3)	15.8	30.0	54.2	
Smoking	No	1196 (57.5)	16.6	30.7	62.8	.15
	Yes	885 (42.5)	16.9	34.4	48.7	
Drinking	No	1303(62.6)	15.8	31.3	52.9	.08
	Yes	778 (37.4)	18.3	33.8	47.9	
ECOG performance status	0	1009 (48.5)	24.6	37.5	38.0	<.001
	1	918 (44.1)	9.8	29.5	60.7	
	2	118 (5.7	5.1	16.1	78.8	
	3	31 (1.5)	12.9	6.5	80.6	
	4	5 (0.2)	0	20.0	80.0	
Stage	1	200 (9.6)	24.0	34.0	42.0	<.001
	2	353 (17.0)	20.7	29.5	49.9	
	3	541 (26.0)	18.1	34.2	47.7	
	4	987 (47.4)	13.1	31.8	55.1	
Primary cancer site	Head and neck	531 (25.5)	20.5	33.0	46.5	<.001
	Esophagus	183 (8.8)	10.4	40.4	49.2	
	Thorax	71 (3.4)	19.7	19.7	60.6	
	Breast	102 (4.9)	28.4	34.3	37.3	
	Stomach or small bowel	316 (15.2)	19.0	32.6	48.4	
	Pancreas	169 (8.1)	7.1	42	50.9	
	Biliary	135 (6.5)	12.6	32.6	54.8	
	Liver	62 (3.0)	11.3	29.0	59.7	
	Colorectal	286 (13.7)	18.2	28.3	53.5	
	Hematologic	148 (7.1)	12.8	29.1	58.1	
	Genitourinary	55 (2.6)	10.9	20.0	69.1	
	Others	23 (1.1)	17.4	8.7	73.9	

Abbreviation: ECOG, Eastern Cooperative Oncology Group.

### Tumor-specific variations in frailty dimensions

Table 2 details the prevalence of impairments across frailty dimensions by primary tumor site. Overall, malnutrition was the most prevalent impairment and affected 59.5% of the patients, followed by comorbidity and polypharmacy. In contrast, social support deficits and falls were the less commonly impaired.

**Table 2.** Impairment for each frailty dimension according to primary tumor site.

Cancer site (number of patients), %^a^	Malnutrition	Comorbidity	Polypharmacy	Functional decline	Cognition	Mood	Social support	Falls
Overall (2081)	59.5	28.0	23.9	20.4	16.7	12.5	8.9	3.7
Head and neck (531)	54.2	19.8	28.4	14.7	10.7	14.3	13.0	1.3
Esophagus (183)	74.9	25.7	19.7	14.2	8.7	10.9	9.3	2.2
Thorax (71)	53.5	38.0	38.0	31.0	25.4	15.5	4.2	5.6
Breast (102)	37.3	26.5	15.7	13.7	10.8	16.7	7.8	2.9
Stomach or small bowel (316)	63.6	21.5	14.9	18.7	14.9	11.7	10.1	5.7
Pancreas (169)	73.4	29.0	19.5	20.7	16.6	10.7	5.3	3.6
Biliary (135)	66.7	31.1	17.8	25.2	26.7	8.9	4.4	5.2
Liver (62)	50.0	50.0	33.9	29.0	24.2	14.5	9.7	8.1
Colorectal (286)	55.2	33.9	28.0	23.1	17.5	10.5	8.0	3.8
Hematologic (148)	60.8	38.5	27.0	29.7	22.3	14.9	6.8	6.1
Genitourinary (55)	61.8	34.5	25.5	40.0	45.5	14.5	3.6	1.8
Others (23)	43.5	60.9	34.8	30.4	52.2	4.3	4.3	4.3

a Percentages represent the proportion of patients with impairment in each domain within each cancer site.

Malnutrition was the most prevalent impairment across different tumor sites, particularly for patients with esophageal, pancreatic, and biliary cancers. In contrast, patients with breast cancer exhibited the lowest malnutrition rates. Comorbidities were the most pronounced in patients with liver cancer, whereas polypharmacy was more common in patients with thoracic and liver malignancies. Functional impairment was a significant issue for patients with genitourinary and thoracic cancers, whereas those with head and neck cancer had the lowest rates. Cognitive impairment varied; the highest prevalence was observed in patients with genitourinary cancers and “other” cancer sites. Mood disorders were more common among patients with breast cancer, and were the least prevalent in those with biliary cancer. Social support deficits were most notable in patients with head and neck cancers, whereas falls remained a relatively minor concern across all groups, with the highest rate observed in patients with liver cancer.

### Descriptive trends in frailty dimensions by cancer site

**Figure 3.**
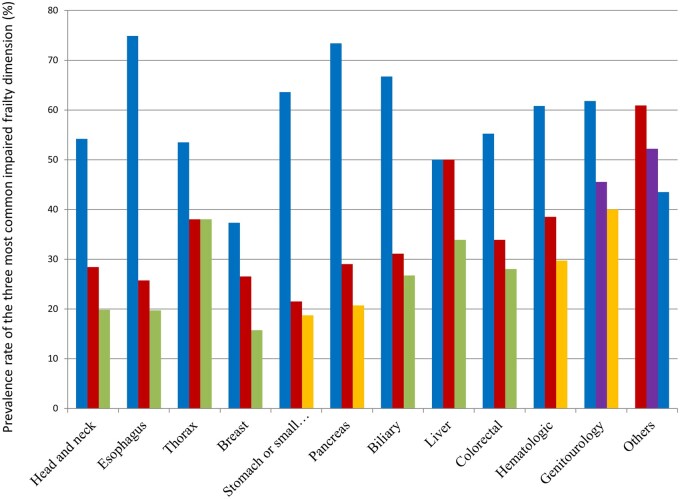
Main frailty domains by cancer sites. Bars represent the percentage of patients with impairments in each domain by frailty group. Blue bar indicates nutrition, red bar indicates comorbidities, green bar indicates polypharmacy, orange bar indicates functional status, and purple bar indicates cognition.

The main frailty factors that emerged as core contributors across different cancer sites were malnutrition, comorbidity, and polypharmacy (Figure 3). Additionally, functional impairment played a prominent role in certain cancer sites, such as stomach/small bowel, pancreatic, hematologic, and genitourinary malignancies. Cognitive impairment was also a major factor for patients with genitourinary and “other” cancer sites.

### Descriptive frailty patterns by age group

**Figure 4.**
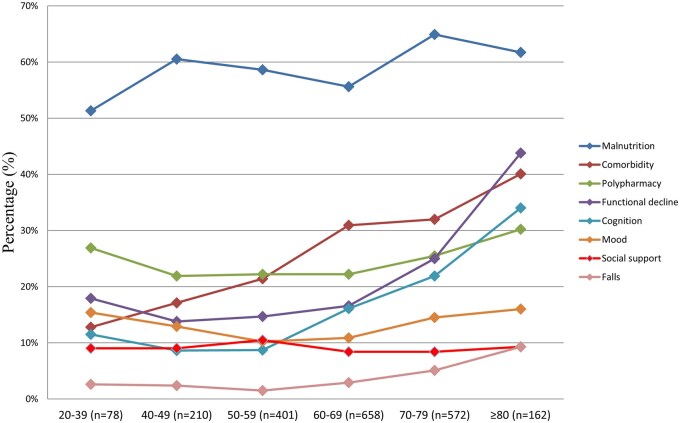
Impairment of each frailty domain according to age groups.

Malnutrition was the most prevalent impairment across all age groups and peaked at 64.9% for the 70-79 years age range, before declining slightly for those aged 80 years and older (Figure 4). Comorbidities demonstrated a pronounced age-related increase, which increased from 12.8% for the 20-39 years group to 40.1% for the 80 years and older cohort. Polypharmacy prevalence also increased with advancing age and reached 30.2% in the oldest group. Among those aged 80 years and older, functional impairment and cognitive impairment exhibited the steepest increases; 43.8% experienced functional impairment; and 34.0% demonstrated cognitive impairment. Mood disturbance remained relatively consistent and peaked at 16.0% in the oldest cohort. Social support deficits remained low and stable across all age groups, which ranged from 8.4% to 10.5%. Although falls were the least common, they showed a noticeable increase with age, which increased from 2.6% in the youngest group to 9.3% in the oldest group.

### Frailty and unplanned hospitalizations

Among the entire cohort, 36.6% of the patients experienced unplanned hospitalizations within 90 days. Patients in the fit group had the lowest hospitalization rate (25.9%), those in the pre-frail group had a moderately higher rate (34.4%), and those in the frailty group had the highest rate (41.5%) (*P* < .001). In the univariate analysis, the OR was 1.51 (95% CI 1.13-2.01, *P* = .005) for the pre-frail and fit groups comparison and 2.04 (95% CI 1.56-2.67, *P* < .001) for the frail and fit groups comparison. In the adjusted logistic regression model, both pre-frail (adjusted OR 1.44, 95% CI 1.06-1.94, *P* = .018) and frail patients (adjusted OR 2.07, 95% CI 1.55-2.76, *P* < .001) had a significantly higher risk of unplanned hospitalization compared to fit individuals. After excluding 108 patients (5.2%) who died within 90 days of treatment initiation, frailty remained independently associated with unplanned hospitalization. The adjusted OR for pre-frail patients was 1.39 (95% CI 1.03-1.89, *P* = .034), and for frail patients, 2.00 (95% CI 1.49-2.67, *P* < .001), confirming the robustness of the association (Table S4). In a sensitivity analysis including ECOG performance status and nutritional status as additional covariates, the association between frailty and unplanned hospitalization remained significant (pre-frail adjusted OR 1.43, 95% CI 1.06-1.93, *P* = .019; frail adjusted OR 2.04, 95% CI 1.54-2.71, *P* < .001).

The OR for unplanned hospitalization for different primary tumor sites stratified by frailty status is shown in Table 3. Using the fit group with the lowest risk of unplanned hospitalizations as the reference group, a general trend emerged: unplanned hospitalizations progressively increased as frailty status worsened, regardless of the primary tumor site. Notably, certain cancer sites, such as head and neck, stomach or small bowel, pancreas, and genitourinary, exhibited notably higher ORs for frailty-related outcomes than other cancer sites. In multivariable models adjusted for age, sex, marital status, and tumor stage, frailty remained an independent predictor in several sites, most notably head and neck cancer (adjusted OR 2.26, 95% CI 1.36-3.76; *P* = .002) and stomach or small-bowel cancer (adjusted OR 2.57, 95% CI 1.05-6.30; *P* = .039). Although the pre-frail group showed a consistent 1.3- to 1.9-fold elevation in odds across most cancer sites, these estimates did not reach statistical significance.

**Table 3.** Odds ratios for 90-day unplanned hospitalization by frailty status and cancer site.

Cancer site (number of patients)	Univariate analysis					Multivariate analysis^a^				
	Fit (reference)	Pre-frail OR (95% CI)	*P*	Frail OR (95% CI)	*P*	Fit (reference)	Pre-frail Adjusted OR (95% CI)	*P*	Frail Adjusted OR (95% CI)	*P*
Head and neck (531)	1	1.43 (0.83-2.45)	.194	2.40 (1.45-3.96)	.001	1	1.39 (0.81-2.39)	.24	2.26 (1.36-3.76)	.002
Esophagus (183)	1	0.89 (0.32-2.47)	.82	1.72 (0.63-4.68)	.29	1	0.96 (0.34-2.71)	.94	1.99 (0.71-5.57)	.19
Thorax (71)	1	1.00 (0.22-4.47)	.99	1.55 (0.45-5.35)	.49	1	1.43 (0.27-7.64)	.67	2.35 (0.54-10.2)	.25
Breast (102)	1	0.79 (0.23-2.79)	.72	1.99 (0.64-6.12)	.23	1	0.56 (0.14-2.29)	.42	1.51 (0.43-5.39)	.52
Stomach or small bowel (316)	1	1.94 (0.77-4.88)	.16	2.68 (1.13-6.38)	.026	1	1.90 (0.74-4.84)	.18	2.57 (1.05-6.30)	.039
Pancreas (169)	1	1.53 (0.38-6.19)	.55	2.38 (0.60-9.39)	.22	1	1.89 (0.44-8.15)	.39	2.74 (0.65-11.6)	.17
Biliary (135)	1	0.78 (0.25-2.40)	.66	1.07 (0.37-3.06)	.91	1	0.74 (0.23-2.39)	.61	1.05 (0.35-3.17)	.93
Liver (62)	1	1.25 (0.19-8.44)	.82	0.80 (0.13-4.88)	.81	1	0.93 (0.07-12.2)	.96	1.36 (0.12-16.1)	.81
Colorectal (286)	1	2.11 (0.82-5.41)	.12	1.57 (0.64-3.82)	.32	1	1.66 (0.60-4.62)	.33	1.34 (0.52-3.71)	.51
Hematologic (148)	1	2.75 (0.85-8.85)	.09	1.11 (0.41-3.05)	.84	1	2.87 (0.79-10.4)	.11	2.12 (0.67-6.75)	.20
Genitourinary (55)	1	2.75 (0.74-103)	.09	2.57 (0.91-80.9)	.06	1	10.9 (0.74-159)	.08	6.20 (0.59-64.9)	.13
Others (23)	1	1.00 (1.00-1.00)	.99	1.80 (0.20-16.5)	.59	1	1.00 (1.00-1.00)	.99	1.22 (0.25-13.1)	.89

a Models adjusted for age, sex, marital status, and tumor stage.

Abbreviation: OR, odds ratio.

In patients aged <65 years, frailty, but not pre-frailty, was significantly associated with increased risk of unplanned hospitalization (adjusted OR 1.81, 95% CI 1.24-2.64; *P* = .002). Among patients aged ≥65 years, both pre-frailty and frailty were significantly associated with hospitalization (adjusted ORs 2.29 and 3.31, respectively; both *P* ≤ .001) (Table S3). Figure S2 (see online supplementary material for a color version of this figure) highlights that malnutrition predominated in younger patients, while older adults more frequently experienced functional decline, comorbidity, and cognitive impairment.

## Discussion

This study examined frailty prevalence and its associated factors across different cancer sites and age groups in a Taiwanese adult population with cancer. Notably, inclusion of the entire adult age continuum revealed that frailty is not confined to geriatric patients; almost half of individuals aged 20-39 years were frail, and their risk of unplanned hospitalization mirrored or exceeded that of older cohorts. These findings corroborate our earlier cohort studies,^22–25^ which likewise demonstrated that disease burden and treatment toxicity can precipitate frailty phenotypes even in younger Taiwanese adults with cancer. Consequently, early frailty screening should be considered even in ostensibly “non-older” patients to allow timely intervention before irreversible decline occurs. These findings indicated that malnutrition, comorbidities, and polypharmacy were the most common contributors to frailty across cancer sites. Frailty rates varied greatly by cancer sites, with higher rates for genitourinary and thoracic cancers and lower rates for breast, esophageal, and stomach/small bowel cancers. Furthermore, frailty prevalence increased significantly with advancing age, with the highest rates observed in those aged 80 years and older. Variations in frailty prevalence across different cancer sites and age groups likely reflected the unique metabolic, functional, and cognitive challenges posed by various cancer sites, their respective treatments, and underlying aging processes.^29^^,^^30^ The link between frailty and hospitalizations emphasizes the importance of frailty assessment in cancer care because it may help identify patients with high risk who may benefit from targeted interventions to prevent adverse outcomes.

Frailty affected 51% of our cohort, underscoring its heavy burden among Taiwanese patients with cancer^18–25^ and mirroring the elevated rates documented in oncology populations worldwide, especially in older adults.^3–8^ This prevalence exceeds the median 42% (range 6%-86%) reported in a large Western systematic review,^5^ a difference that may stem from variations in frailty definitions, underlying health status, and healthcare-system or cultural contexts. By contrast, our findings are consistent with recent Taiwanese data,^18–25^ suggesting that regional factors, such as higher baseline rates of malnutrition or comorbidity, may shape frailty patterns. The multidimensional character of frailty was clear in our study, with malnutrition, comorbidities, and polypharmacy emerging as key contributors across tumor sites and age groups.^27^^,^^28^ The observed variability in frailty across cancer sites may reflect differences in baseline symptom burden, disease trajectory, and the extent to which cancer symptoms contribute to functional impairment prior to treatment rather than a need for site-specific frailty definitions.^31^ For example, gastrointestinal or hematologic malignancies may be associated with early nutritional and physical impairments, while genitourinary cancers may present with cognitive or functional limitations in older patients.

The results of this study provide several insights about the demographic factors associated with frailty in patients with cancer. The findings align with previous research indicating that frailty is more common among older adults, those with lower socioeconomic status, and individuals with advanced cancer.^3^^,^^15^^,^^32–34^ Age is a key driver of frailty and its prevalence increases significantly with older patients.^15^ The data from this study showed a nearly 30 percentage point rise in frailty prevalence between the youngest age group (20-39 years) and the oldest age group (80 years and older). The oldest patients exhibited the highest prevalence across several key frailty dimensions, including malnutrition, comorbidity, polypharmacy, functional impairment, and cognitive impairment. This is consistent with the understanding that frailty is a geriatric syndrome characterized by a decline in physiological reserves, unmet basic resource needs, and increased vulnerability to adverse health outcomes.^35^^,^^36^ The clinical expression and prognostic value of frailty appear to differ by age. As shown in Figure S2 (see online supplementary material for a color version of this figure), malnutrition was more prevalent in younger patients, while older adults were more commonly affected by functional decline, comorbidities, and cognitive impairment. Despite these differences, frailty remained an independent predictor of early hospitalization across both age groups. These findings support the universal application of frailty screening in oncology care, with tailored interventions based on age-­specific frailty profiles.

The sex differences for frailty prevalence were relatively small, with a slightly higher prevalence among women than among men. This aligns with previous studies that suggest that women may have a higher risk for frailty, potentially due to factors, such as differences in body composition, hormonal changes, and sociocultural dynamics.^15^^,^^36^^,^^37^ The higher rates of frailty observed in the unmarried, unemployed, and less-educated groups likely reflect their increased vulnerability to the physical, cognitive, and social impairments that contribute to frailty.^38^ Understanding the factors that contribute to frailty in patients with cancer is crucial for developing targeted interventions.

Certain cancer sites, such as genitourinary and thoracic cancers, exhibited a higher frailty prevalence than other tumor sites. This may be attributed to the distinct metabolic, functional, and cognitive challenges posed by these cancer sites.^28^^,^^39^^,^^40^ Conversely, patients with breast, esophageal, and stomach/small bowel cancers had lower frailty rates, potentially reflecting differences in disease burden and underlying patient characteristics. Tumor stage was also a significant factor; the patients with more advanced disease stages had greater frailty prevalence.^41^^,^^42^ Further research is required to elucidate the complex interplay between specific tumor types, disease characteristics, and frailty.

The observed associations between frailty and adverse hospitalization outcomes underscore the critical importance of comprehensive frailty assessment as a routine component of cancer care.^6^ Identifying patients at high-risk for frailty is crucial because it enables the implementation of targeted interventions to mitigate poor clinical outcomes.^43^ Consistent with the growing body of evidence, our analysis revealed that the risk of unplanned hospitalizations was significantly higher among patients with cancer and frailty than among their fitter counterparts across various tumor types.^44^ Notably, certain cancer sites, such as head and neck, stomach/small bowel, pancreas, and genitourinary malignancies, exhibited particularly elevated unplanned hospitalizations associated with frailty. These findings highlight the need to prioritize frailty assessment and development of tailored interventions to optimize the management of high-risk patients with cancer.^45^

Importantly, our findings also highlight that the pre-frail group, often overlooked in clinical decision-making, had a significantly increased risk of unplanned hospitalization compared to fit patients. This intermediate group may represent a critical window for early intervention, where timely assessment and tailored supportive measures (eg, nutritional optimization, medication review, and mobility preservation) could prevent further decline into frailty. Recognizing and addressing pre-frailty in routine oncology care may enhance outcomes through preventative strategies before irreversible deterioration occurs.

The strength of this study is its large population-based sample, which allowed for detailed analyses across age groups and cancer sites. To our knowledge, this study provides the first detailed characterization of frailty dimensions across different cancer sites and age groups in a Taiwanese population. These findings can provide information for the development of targeted frailty assessments and intervention strategies to improve the care and outcomes of older adults with cancer.

This study also has several limitations. First, this study was conducted in Taiwan, where the National Health Insurance program provides universal healthcare access with minimal out-of-pocket costs. This system may facilitate more timely hospital admissions for symptom management or supportive care, potentially influencing the observed rates of unplanned hospitalization. In contrast, in settings with limited insurance coverage or higher patient cost-sharing, thresholds for hospitalization may differ. Therefore, while the strong association between frailty and hospitalization is likely generalizable, the absolute rates and patterns of hospitalization observed in this study may not directly apply to healthcare systems with different financing or access models. Second, our CGA-based, cutoff-based frailty thresholds—though drawn from prior studies^25^^,^^28^—may not capture the full spectrum of functional heterogeneity across cancer sites or age groups and could misclassify borderline cases. Third, potential interactions among frailty domains were not modeled, and despite multivariable adjustment residual confounding from unmeasured factors (eg, caregiver support, comorbidity severity beyond CCI) remains possible; exclusion of ECOG performance status and nutritional status from the primary model for collinearity was mitigated by sensitivity analyses that confirmed the robustness of our findings. We also did not perform formal proportional-odds diagnostics because no ordinal models were used, but future studies could treat frailty as an ordinal exposure and test these assumptions. Fourth, the linked EMRs of our 3 centers did not capture hospitalizations at outside facilities, likely causing a modest under-estimation of absolute rates. Fifth, our primary analysis treated death as a non-event, even though it is a conceptual competing risk for unplanned hospitalization. We therefore performed a sensitivity analysis that excluded patients who died within 90 days; the association between frailty and hospitalization remained robust. Nevertheless, the lack of precise time-to-event data and cause-specific mortality coding prevented us from applying more sophisticated competing-risk approaches. Finally, the absence of a universally standardized frailty assessment hampers direct comparison across studies. Future research should adopt harmonized tools, incorporate broader social determinants and patient-reported measures, and track long-term outcomes to deepen understanding of frailty-related hospitalizations.

## Conclusion

This large population-based study provides a comprehensive assessment of frailty prevalence and its associated factors among older adults with cancer in Taiwan. These findings demonstrated that frailty was highly prevalent, particularly among the oldest patients and those with advanced cancer stages, lower socioeconomic status, and certain cancer sites. The robust correlation between frailty and adverse hospitalization outcomes emphasizes the importance of routinely assessing frailty as an integral component of cancer care. These findings can provide a crucial first step in developing tailored frailty assessments and intervention strategies for high-risk populations with cancer that can potentially improve outcomes and optimize the delivery of personalized patient-centered care.

## Supplementary Material

oyaf234_Supplementary_Data

## Data Availability

The datasets used and/or analyzed in the current study are available from the corresponding author upon reasonable request.
