# Supplementary material for: Prevalence and impact of frailty on unplanned hospitalizations among adult patients with cancer in Taiwan
Source: Oncologist. 2025 Jul 26;30(9):oyaf234. doi: 10.1093/oncolo/oyaf234 (PMC12422316; doi:10.1093/oncolo/oyaf234)

Supplementary Table 1. Measurements and cut-off points for the frailty dimension of the comprehensive geriatric assessment

| Dimension | Measures | Score range | Cutoff value |
| --- | --- | --- | --- |
| Nutrition | MNA-SF | 0–14 | ≤ 11 |
| Comorbidity | CCI^a^ | 0–33 | ≥ 2 |
| Functional status | Barthel Index (ADL) or  Lawton (IADL) scale | 0–100  0–8 | ADL < 100 or  IADL ≤ 7 |
| Polypharmacy | Number of medications | 0–∞ | ≥ 5 |
| Mood | GDS-4 | 0–4 | ≥ 2 |
| Cognition | Mini-Mental State Examination | 0–30 | ≤ 23 |
| Social support | Living alone | 0–1 | 1 |
| Falls | Number of falls | 0–∞ | ≥ 2 |

ADL, activities of daily living; CCI, Charlson Comorbidity Index; IADL, instrumental activities of daily living; GDS-4, Geriatric Depression Scale 4-item; MNA-SF, Mini Nutritional Assessment Short Form.

^a^A modified CCI excluding scores for patient age and cancer diagnosis was used in this study.

Supplementary Table 2. Patient characteristics stratified by cancer site

| Variables | Head and neck (n=531) | Esophagus (n=183) | Thorax (n=71) | Breast (n=102) | Stomach or small bowel (n=316) | Pancreas (n=169 | Biliary (n=135) | Liver (n=62) | Colorectal (n=286) | Hematologic (n=148) | Genitourinary (n=55) | Others (n=23) |
| --- | --- | --- | --- | --- | --- | --- | --- | --- | --- | --- | --- | --- |
| Median age (range) | 54 (24-86) | 56 (28-84) | 64 (27-81) | 67 (36-84) | 70 (32-94) | 69 (38-85) | 70 (43-100) | 71 (44-91) | 71 (39-99) | 67 (22-96) | 71 (20-85) | 65 (21-83) |
| Male | 86.3% | 90.2% | 57.7% | 1.0% | 64.1% | 60.9% | 53.3% | 67.7% | 61.2% | 60.8% | 69.1% | 78.3 |
| Married | 78.4% | 79.8% | 83.1% | 76.5% | 83.2% | 87.0% | 85.2% | 72.6% | 75.2% | 75.7% | 81.8% | 87.0 |
| Stage 1 | 3.2% | 2.2% | 8.5% | 14.7% | 21.2% | 3.6% | 4.4% | 33.9% | 15.4% | 6.8% | 7.3% | 0.0% |
| Stage 2 | 13.4% | 13.1% | 5.6% | 55.9% | 14.6% | 10.1% | 15.6% | 29.0% | 19.9% | 20.9% | 10.9% | 4.3% |
| Stage 3 | 16.8% | 57.4% | 22.5% | 11.8% | 34.2% | 18.3% | 23.0% | 17.7% | 36.0% | 11.5% | 29.1% | 8.7% |
| Stage 4 | 66.7% | 27.3% | 63.4% | 17.6% | 30.1% | 68.0% | 57.0% | 19.4% | 28.7% | 60.8% | 52.7% | 87% |
| ECOG of 0 | 47.6% | 45.4% | 28.2% | 69.6% | 56.6% | 36.7% | 42.2% | 59.7% | 62.9% | 27.0% | 38.2% | 26.1% |
| ECOG of 1 | 50.1% | 51.4% | 50.7% | 23.5% | 36.4% | 47.9% | 47.4% | 37.1% | 30.1% | 58.8% | 47.3% | 69.2% |
| ECOG of 2 ~4 | 2.3% | 3.3% | 21.1% | 6.9% | 7.0% | 15.4% | 10.4% | 3.2% | 7.0% | 14.2% | 14.5% | 4.7% |
| Operation* | 0.2% | 0.0% | 2.8% | 14.7% | 63.0% | 8.3% | 17.0% | 82.3% | 62.2% | 0.0% | 5.5% | 4.3% |
| Chemotherapy* | 13.9% | 26.2% | 97.2% | 85.3% | 37.0% | 91.7% | 83.0% | 17.7% | 37.8% | 100.0% | 94.5% | 87.0% |
| CCRT* | 85.9% | 73.8% | 0.0% | 0.0% | 0.0% | 0.0% | 0.0% | 0.0% | 0.0% | 0.0% | 0.0% | 8.7% |

* main treatment after frailty assessment

ECOG, Eastern Cooperative Oncology Group; CCRT, concurrent chemoradiotherapy

Supplementary Table 3. Multivariable logistic regression models for unplanned hospitalization stratified by age group

| Age group | Frailty | Adjusted odds ratio* | 95% confidence interval | p-value |
| --- | --- | --- | --- | --- |
| <65 | Fit | 1 (reference) |  |  |
|  | Pre-frail | 1.15 | 0.78-1.70 | 0.48 |
|  | Frail | 1.81 | 1.24-2.64 | 0.002 |
| ≥65 | Fit | 1 (reference) |  | 0.004 |
|  | Pre-frail | 2.29 | 1.42-3.71 | 0.001 |
|  | Frail | 3.31 | 2.11-5.19 | <0.001 |

* Models adjusted for sex, marital status, and tumor stage.

Supplementary 4. Logistic regression analysis for unplanned hospitalisation in full model (n=2081) and excluding patients who died within 90 days (remaining patient number=1973).

| Patient population | Frailty | Unplanned hospitalization rate | Adjusted odds ratio* | 95% confidence interval | p-value |
| --- | --- | --- | --- | --- | --- |
| Full model (n=2081) | Fit | 25.9% | 1 (reference) |  |  |
|  | Pre-frail | 34.4% | 1.44 | 1.06-1.94 | 0.018 |
|  | Frail | 41.5% | 2.07 | 1.55-2.76 | <0.001 |
| Excluding patients who died within 90 days (n=1973) | Fit | 24.9% | 1 (reference) |  | 0.004 |
|  | Pre-frail | 32.3% | 1.39 | 1.03-1.89 | 0.034 |
|  | Frail | 38.9% | 2.00 | 1.49-2.67 | <0.001 |

* adjusted for age, sex, marital status, and tumor stage.

Supplementary Figure 1. Directed acyclic graph used to identify confounding variables in the relationship between frailty and 90-day unplanned hospitalization.


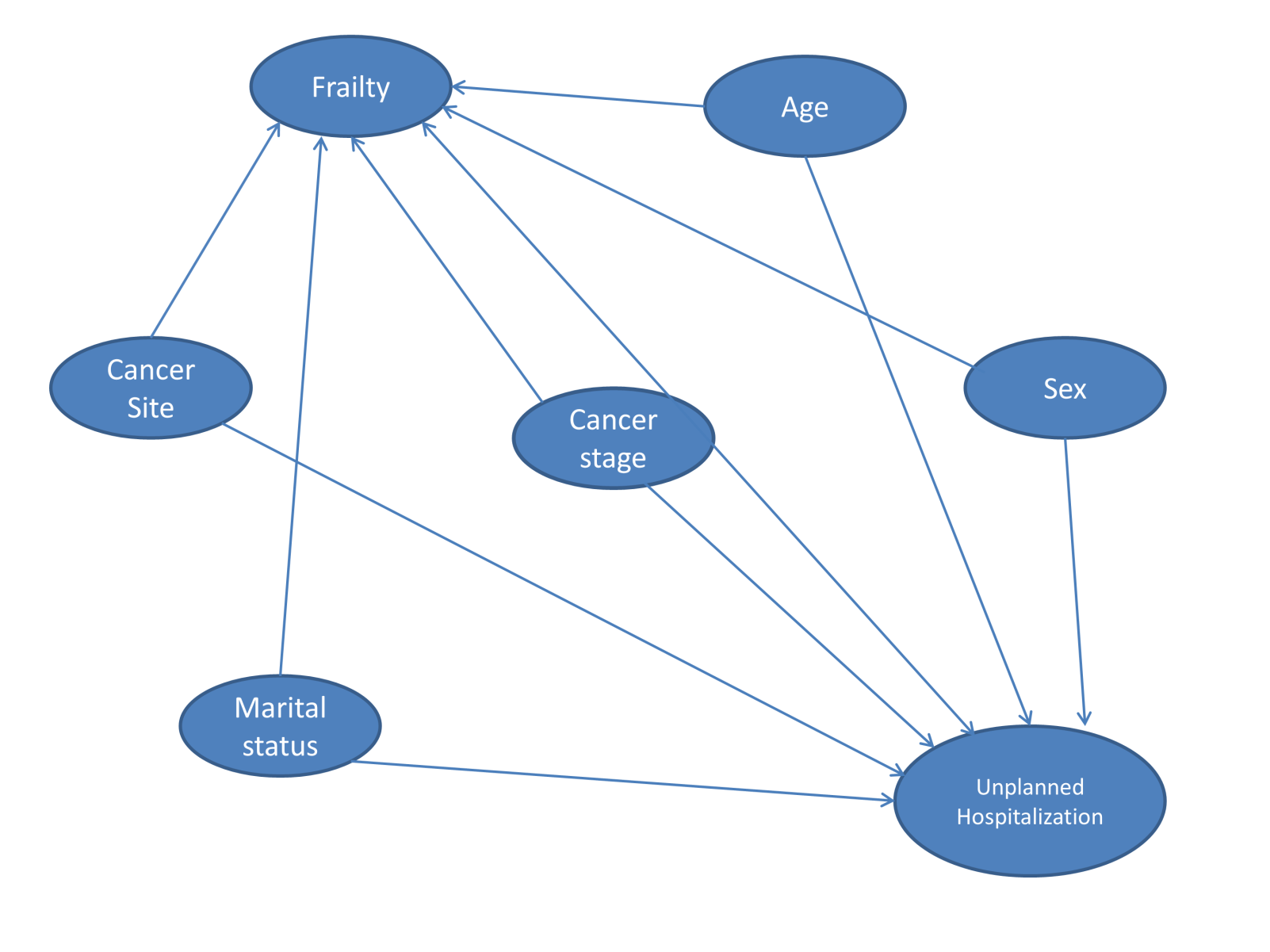


Supplementary Figure 2. Prevalence of impaired frailty dimension stratified by age group


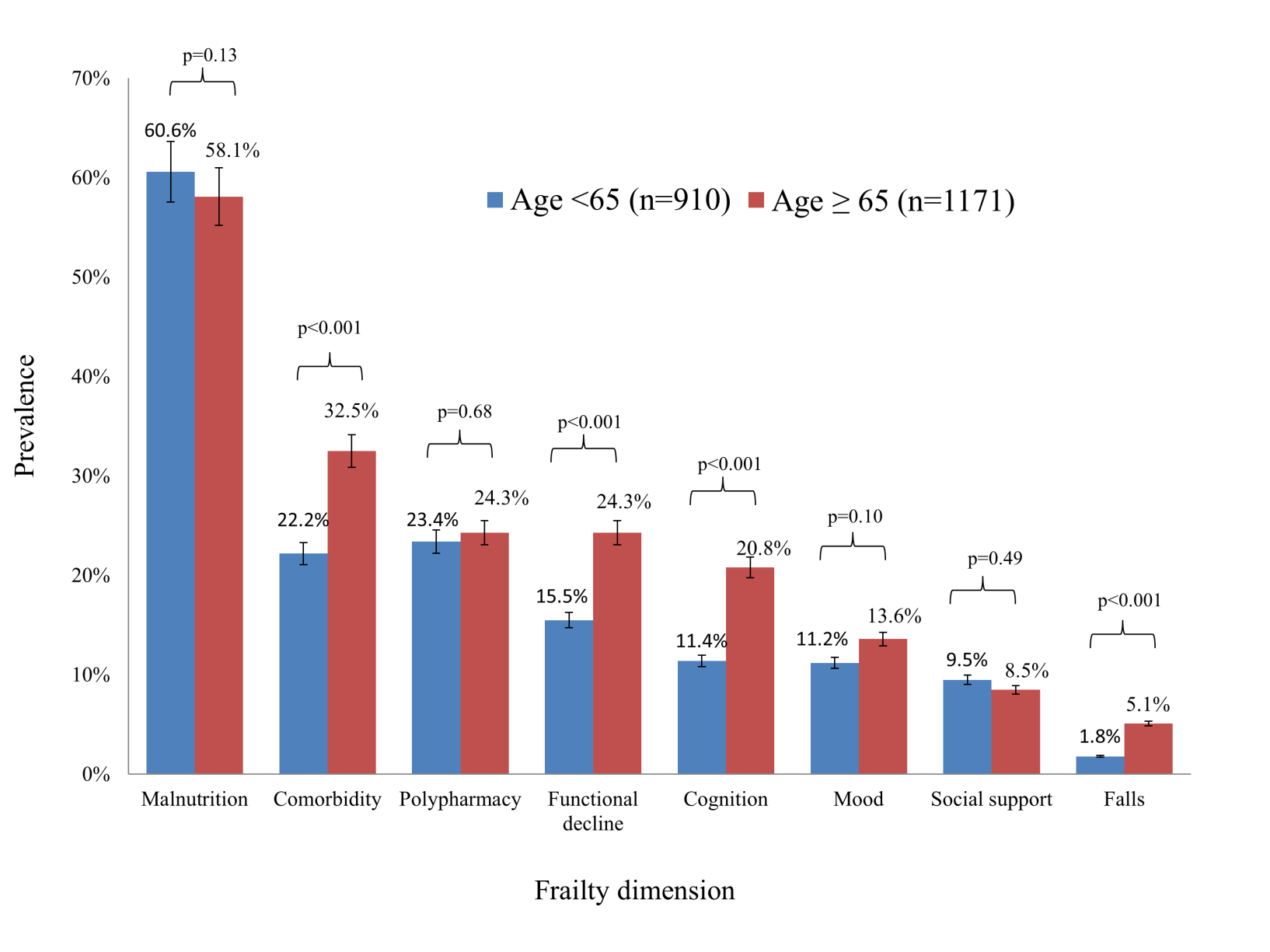

Supplement: oyaf234_Supplementary_Data [file oyaf234_supplementary_data.docx]
